# Highly Heterogeneous Excitatory Connections Require Less Amount of Noise to Sustain Firing Activities in Cortical Networks

**DOI:** 10.3389/fncom.2018.00104

**Published:** 2018-12-21

**Authors:** Hisashi Kada, Jun-nosuke Teramae, Isao T. Tokuda

**Affiliations:** ^1^Department of Mechanical Engineering, Ritsumeikan University, Kusatsu-shi, Japan; ^2^Graduate School of Informatics, Kyoto University, Kyoto, Japan

**Keywords:** network firing activity, cortical network, lognormal distribution, excitatory and inhibitory connections, heterogeneity, synaptic noise

## Abstract

Cortical networks both *in vivo* and *in vitro* sustain asynchronous irregular firings with extremely low frequency. To realize such self-sustained activity in neural network models, balance between excitatory and inhibitory activities is known to be one of the keys. In addition, recent theoretical studies have revealed that another feature commonly observed in cortical networks, i.e., sparse but strong connections and dense weak connections, plays an essential role. The previous studies, however, have not thoroughly considered the cooperative dynamics between a network of such heterogeneous synaptic connections and intrinsic noise. The noise stimuli, representing inherent nature of the neuronal activities, e.g., variability of presynaptic discharges, should be also of significant importance for sustaining the irregular firings in cortical networks. Here, we numerically demonstrate that highly heterogeneous distribution, typically a lognormal type, of excitatory-to-excitatory connections, reduces the amount of noise required to sustain the network firing activities. In the sense that noise consumes an energy resource, the heterogeneous network receiving less amount of noise stimuli is considered to realize an efficient dynamics in cortex. A noise-driven network of bi-modally distributed synapses further shows that many weak and a few very strong synapses are the key feature of the synaptic heterogeneity, supporting the network firing activity.

## 1. Introduction

Intrinsic neuronal activities in the cortical network are inherently noisy (Tomko and Crapper, 1974; Softky and Koch, 1993; Shadlen and Newsome, 1994; Faisal et al., 2008; Stiefel et al., 2013). The so-called spontaneous irregular firings have been characterized by extremely low firing frequency (Hromádka et al., 2008; Mizuseki and Buzsáki, 2013), high-amplitude membrane potential fluctuations (Wilson and Kawaguchi, 1996; Destexhe et al., 2001), persistent UP (depolarized) state of membrane potential (Steriade et al., 2001; Destexhe et al., 2003; Shu et al., 2003a), and sensitivity to perturbations (London et al., 2010). They have been observed in cortical cell cultures (Gross et al., 1982; Plenz and Aertsen, 1996; Marom and Shahaf, 2002), brain slices (Mao et al., 2001; Shu et al., 2003b), and *in vivo* (Timofeev et al., 2000) even in the absence of external stimuli. Such randomness is considered to play a key role in various computations in the cortex, ranging from sensory perception (Arieli et al., 1996; Tsodyks et al., 1999), working memory (Fuster, 1995; Wang, 2002; Compte, 2006), dynamical switching of cortical states (Kenet et al., 2003) to information propagation (Destexhe and Contreras, 2006; Kumar et al., 2010). Although detailed cortical microcircuitry is yet to be explored, theoretical models have been developed to elucidate the electrophysiological mechanisms that underlie the spontaneous firing. The modeling approaches can be roughly classified into two types: externally driven network dynamics vs. self-sustained network dynamics. In the externally driven network, individual neurons as well as their network state are quiescent when no external input is provided. The network dynamics is activated by external stimuli such as background noise inputs, which may represent, e.g., variability of presynaptic discharges of individual cells (Destexhe and Contreras, 2006). Alternatively, existence of a certain portion of endogenously active cells within a network of quiescent cells may also act as external input to maintain the ongoing firing state (Latham et al., 2000a,b). In the modeling type of self-sustained network dynamics, on the other hand, interaction between a network of cortical neurons plays an essential role for maintaining the intrinsic firing activities. In random networks of leaky integrate-and-fire neurons, balance between excitatory and inhibitory activities is shown to be the key to sustain asynchronous irregular dynamics (van Vreeswijk and Sompolinsky, 1996; Brunel, 2000) even without external stimuli (Vogels and Abbott, 2005; Kumar et al., 2008). Modeling the statistical features of synaptic strengths of cortical neurons (Song et al., 2005; Lefort et al., 2009; Avermann et al., 2012; Buzsáki and Mizuseki, 2014) enabled asynchronous irregular firing in a biologically plausible parameter range. Teramae et al. (2012) studied a network of leaky integrate-and-fire neuron model with lognormally distributed excitatory postsynaptic potentials (EPSPs) and showed analytically that low-frequency firings of few Hz can be generated without external stimuli. Ikegaya et al. (2013) observed lognormal distribution in the amplitudes of unitary excitatory postsynaptic conductance in rat hippocampal CA3 pyramidal cells. Lognormally distributed excitatory-to-excitatory synapses, fitted from the experimental data, were implemented into the recurrent network model, which realized long-tailed distribution of firing rates and infrequent spontaneous firings (< 2 Hz) without external stimuli. Kriener et al. (2014) studied a network of strong excitatory-to-excitatory synapses and showed that bistability of quiescent state and moderate firing state is the key determinant for the onset and the lifetime of self-sustained activity states.

As a combined situation of the two types, cooperative dynamics between the network of long-tailed synaptic distribution and the external noise stimuli has not yet been thoroughly investigated. Kriener et al. (2014) simulated external *Poisson* processes to drive such network and reported an amplification of the input spike correlations and the spike irregularity, leading to fluctuations of a large population of neurons. They, however, focused on the noise input not as the main source of sustaining the network dynamics. Of particular interest in the present study is that the noise may enhance subthreshold state of the intrinsically active network, leading to the onset of self-sustained neuronal firings. Since the noise requires an energy resource (Laughlin et al., 1998; Attwell and Laughlin, 2001; Lennie, 2003), it is biologically more efficient to maintain the network firing activities with less amount of noise. To distinguish the present approach from the preceding studies (Vogels and Abbott, 2005; Kumar et al., 2008; Teramae et al., 2012; Ikegaya et al., 2013; Kriener et al., 2014), which discussed spontaneous firings without assuming any external stimulus, we refer to low-frequency irregular firings observed in our noise-driven system as “network firing activities.” We numerically detect the minimal level of noise that gives rise to the network firing activities and compared such levels between networks of lognormally and normally distributed EPSPs. Our study shows that the noise required to generate the network firings is strongly reduced in the network of lognormally distributed EPSPs. Examination of the network of bi-modally distributed EPSPs further clarifies that essential mechanism of the network can be determined only by a pair of one weak and one strong EPSP values and their balance. Our results suggest a strong advantage of the heterogeneous excitatory-to-excitatory connections that realize robust and efficient operation of the cortical network.

The rest of the paper is organized as follows. In section 2, a network of integrate-and-fire neurons is introduced as a mathematical model for the cortical network. Quantity to measure the level of synchronized firings is also introduced. In section 3, the effect of dynamical noise on the network of lognormally distributed EPSPs is studied in comparison to a network of normally distributed EPSPs. By introducing *Bernoulli* distribution of binary variables, which represent weak and strong synapses, to the noise-driven neural network, the key statistical feature is highlighted for the heterogeneous distribution of the EPSPs. The final section is devoted to our results summary and discussions on future problems of the cortical networks.

## 2. Methods

### 2.1. Model of a Single Neuron

The following conductance-based leaky integrate-and-fire neuron was utilized to represent the dynamics of individual neuron

(1) $$\begin{array}{r} \frac{dv}{dt}=-\frac{1}{\tau_{m}}\left(v-V_{L}\right)-g_{E}\left(v-V_{E}\right)-g_{I}\left(v-V_{I}\right), \end{array}$$

where *v* stands for membrane potential, τ_*m*_ is a membrane time constant, and *V*_*L*_, *V*_*E*_, and *V*_*I*_ denote reversal potential of leak, excitatory, and inhibitory postsynaptic currents, respectively. The constant values were set to τ_*m*_ = 20 ms for excitatory neurons, τ_*m*_ = 10 ms for inhibitory neurons, *V*_*L*_ = −70 mV, *V*_*E*_ = 0 mV, and *V*_*I*_ = −80 mV.

The excitatory and inhibitory synaptic conductance normalized by the membrane capacitance, *g*_*E*_ and *g*_*I*_, evolves as follows:

(2) $$\begin{array}{r} \frac{dg_{X}}{dt}=-\frac{g_{X}}{\tau_{s}}+\underset{j}{\sum }G_{X,j}\underset{s_{j}}{\sum }\delta \left(t-s_{j}-d_{j}\right),\;X=E,I, \end{array}$$

where the indices *X* = *E* and *X* = *I* are for excitatory and inhibitory conductance, respectively. δ(*t*) represents the delta function, *G*_*X, j*_, *d*_*j*_, and *s*_*j*_ are synaptic weight, delay, and spike timing of synaptic input from the *j*-th neuron, respectively. The decay time constant τ_*s*_ was set to 2 ms for both excitatory and inhibitory conductance. The synaptic delays *d*_*j*_ obey uniform distribution that ranges from *d*_0_−1 to *d*_0_+1 [ms]. The mean synaptic delay was set to *d*_0_ = 2 ms for excitatory-to-excitatory connections and *d*_0_ = 1 ms for other connections. The threshold value for spike generation was set to *V*_*thr*_ = −50 mV, where *v* was reset to *V*_*r*_ = −70 mV after the spiking. The refractory period was set to 1 ms. These parameter setting is based on the one use by Teramae et al. (2012).

### 2.2. Organization of Cortical Network Model

**Figure 1:**
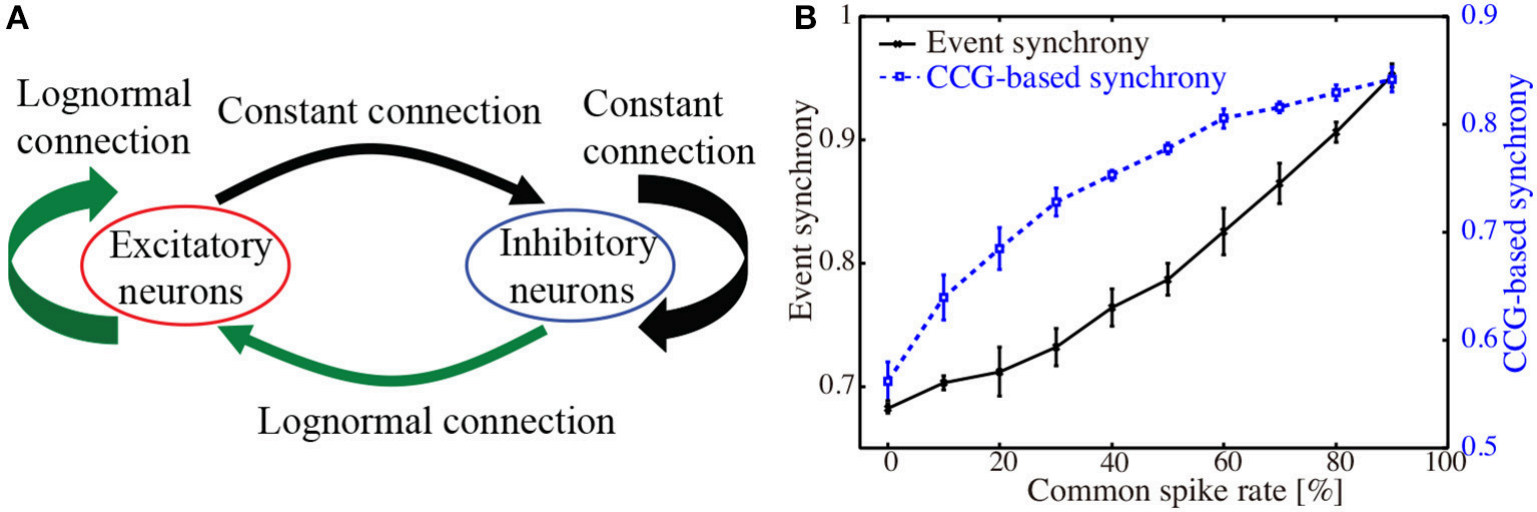
**(A)**. Schematic illustration of the network of excitatory and inhibitory neurons. Lognormally distributed EPSPs were set to excitatory-to-excitatory and inhibitory-to-excitatory connections. Uniform connections were set to other types. *Poisson* spike trains were added externally to all excitatory neurons. **(B)** CCG-based synchronization index and event synchrony computed for 50 sets of *Poisson* spike trains. Percentage of the common spikes included in the individual spike trains was increased from 0 to 90 %.

The network model consisted of 10, 000 excitatory neurons and 2, 000 inhibitory neurons (Figure 1A). Based on the physiological measurements of Song et al. (2005), probability that a pair of excitatory neurons is bidirectionally connected was set to *P*_*bi*_ = 0.0542, while probability that a pair is unidirectionally connected was set to *P*_*uni*_ = 0.123. For excitatory-to-excitatory connections, their synaptic weights *G*_*E, j*∈*E*_ were randomly generated such that the amplitudes of EPSPs *x* measured from the resting membrane potential obey the following lognormal distribution

(3) $$\begin{array}{r} p\left(x\right)=\frac{\exp\left[-{\left(\log x-\mu_{L}\right)}^{2}/2\sigma_{L}^{2}\right]}{\sqrt{2\pi }\sigma_{L}x}. \end{array}$$

μ_*L*_ and σ_*L*_ represent mean and standard deviation of the variable's natural logarithm. As the parameter to control variability of the EPSPs, the value of σ_*L*_ was varied under the condition that the mean EPSP remained the same, i.e., $\mu_{L}+\sigma_{L}^{2}/2=\log\left(0.9\right)$. The case of $\sigma_{L}^{2}=1.0$ reproduces the long-tailed EPSP distribution observed in the experiment of Song et al. (2005). When generating the synaptic weights, any unrealistic value *G*_*E, j*∈*E*_ that gave EPSP amplitude larger than 20 mV was discarded and we selected another value from the distribution.

Following the former study of Teramae et al. (2012), a constant value of *G*_*E, j*∈*I*_ = 0.018 was used for the excitatory-to-inhibitory connections. Since the inhibitory-to-inhibitory synaptic weight was set to be relatively small by Teramae et al. (2012), the weight value was modified as *G*_*I, j*∈*I*_ = 0.018. The corresponding postsynaptic potentials, which were measured from the resting membrane potential of −70 mV (EPSP) and −55 mV (IPSP), were 1.66 and −0.55 mV, respectively. They are within the range of experimentally measured postsynaptic potentials (Tamás et al., 1998; Avermann et al., 2012; Jiang et al., 2015).

It has been shown that lognormal distribution of inhibitory-to-excitatory connections plays an important role of suppressing synchronized neuronal firings especially in a high-frequency firing state (Kada et al., 2016). This setting is consistent with the physiological experiments reporting that inhibitory postsynaptic potentials (IPSPs) are indeed highly heterogeneous in the cortex (Miles and Wong, 1984; Holmgren et al., 2003; Chapeton et al., 2012). Following these studies, the synaptic weights *G*_*I, j*∈*E*_ were generated so that the corresponding IPSPs on excitatory neurons were lognormally distributed as $p\left(x\right)=\frac{\exp\left[-{\left(\log x-\mu_{I}\right)}^{2}/2\sigma_{I}^{2}\right]}{\sqrt{2\pi }\sigma_{I}x}$. The mean and standard deviation of the variable's natural logarithm were set as $\mu_{I}=\log\left(0.52\right)-\sigma_{I}^{2}/2$ and σ_*I*_ = 1.25, respectively. Any unrealistic value of *G*_*I, j*∈*E*_ that gave IPSP amplitude larger than 30 mV was discarded and another value was selected from the distribution.

The connection probabilities of excitatory-to-inhibitory, inhibitory-to-excitatory and inhibitory-to-inhibitory neurons were *P*_*EI*_ = 0.1157, *P*_*IE*_ = 0.3, and *P*_*II*_ = 0.32, respectively. Excitatory-to-excitatory synaptic transmissions failed with a rate *p*_*E*_ = *b*/(*b*+*EPSP*) (*b* = 0.1). This formula has been developed by Teramae et al. (2012) to model the experiment of Lefort et al. (2009), who reported that trial-to-trial variability of large-amplitude EPSPs is very low compared with highly variable responses observed at small-amplitude synaptic connections.

It has been known that presynaptic discharges of individual cells are variable in time and can be represented as background noise inputs (Tomko and Crapper, 1974; Softky and Koch, 1993; Shadlen and Newsome, 1994; Destexhe and Contreras, 2006; Faisal et al., 2008). To take into account such noisy inputs, external *Poisson* spike trains were added to all excitatory neurons. The Euler's method was used to integrate the differential Equations (1),(1) with a time step of 0.01 ms.

### 2.3. Index of Synchronized Firings

We quantified the level of synchronization between spike trains based on the cross-correlogram (CCG). The cross-correlogram CCG has been a standard method for detecting spike synchrony (Logigian et al., 1988; Gray et al., 1989; Datta and Stephens, 1990; Bremner et al., 1991; Engel et al., 1991; Nordstrom et al., 1992). First, from 10, 000 excitatory neurons, 100 neurons were randomly selected. Then, the CCG was drawn as a histogram of spike-time differences (bin size: 1 ms, time lag: ±20 ms) for all pairs of the 100 neurons. In accordance with our previous study (Kada et al., 2016), we define the synchronization index as a normalized height of the peak,

(4) $$\begin{array}{r} SI=\frac{M-A}{M}, \end{array}$$

where *M* and *A* are the maximum and the average of CCG, respectively. The present index quantifies the level of synchrony, since peaks in the CCG indicate occurrences of synchronized firings.

As a quantity to measure distance as well as synchrony between spike trains, various methods have been developed (Victor and Purpura, 1996; van Rossum, 2001; Quian Quiroga et al., 2002; Aronov, 2003; Kreuz et al., 2007, 2009). To validate our approach, the CCG-based synchronization index was compared with the event synchrony (Quian Quiroga et al., 2002), which is one of the simple yet well-established methods to quantify synchronized firings.

As a test data-set, we generated 50 independent *Poisson* spike trains with a time-interval of 20 s. Then, another *Poisson* spike train was generated and included in the 50 spike trains as the common spikes. Frequency of the common spikes ranged from 0 to 9 Hz, while that of the individual spike trains was set to be 10 Hz. Figure 1B shows the results of applying our synchronization index and the event synchrony to these artificial data. As the frequency of the common spikes was increased, the level of synchrony monotonously increased for both CCG-based synchronization index and the event synchrony. The error-bars, obtained from 10 realizations of different data sets, were relatively small. Correlation coefficient between the CCG-based index and the event synchrony was *r* = 0.872 (*p* < 10^−4^), indicating that the present index provides a reliable measure for detecting the synchronized firings.

## 3. Results

### 3.1. Noise-Induced Network Firing in the Network of Lognormally Distributed EPSPs

**Figure 2:**
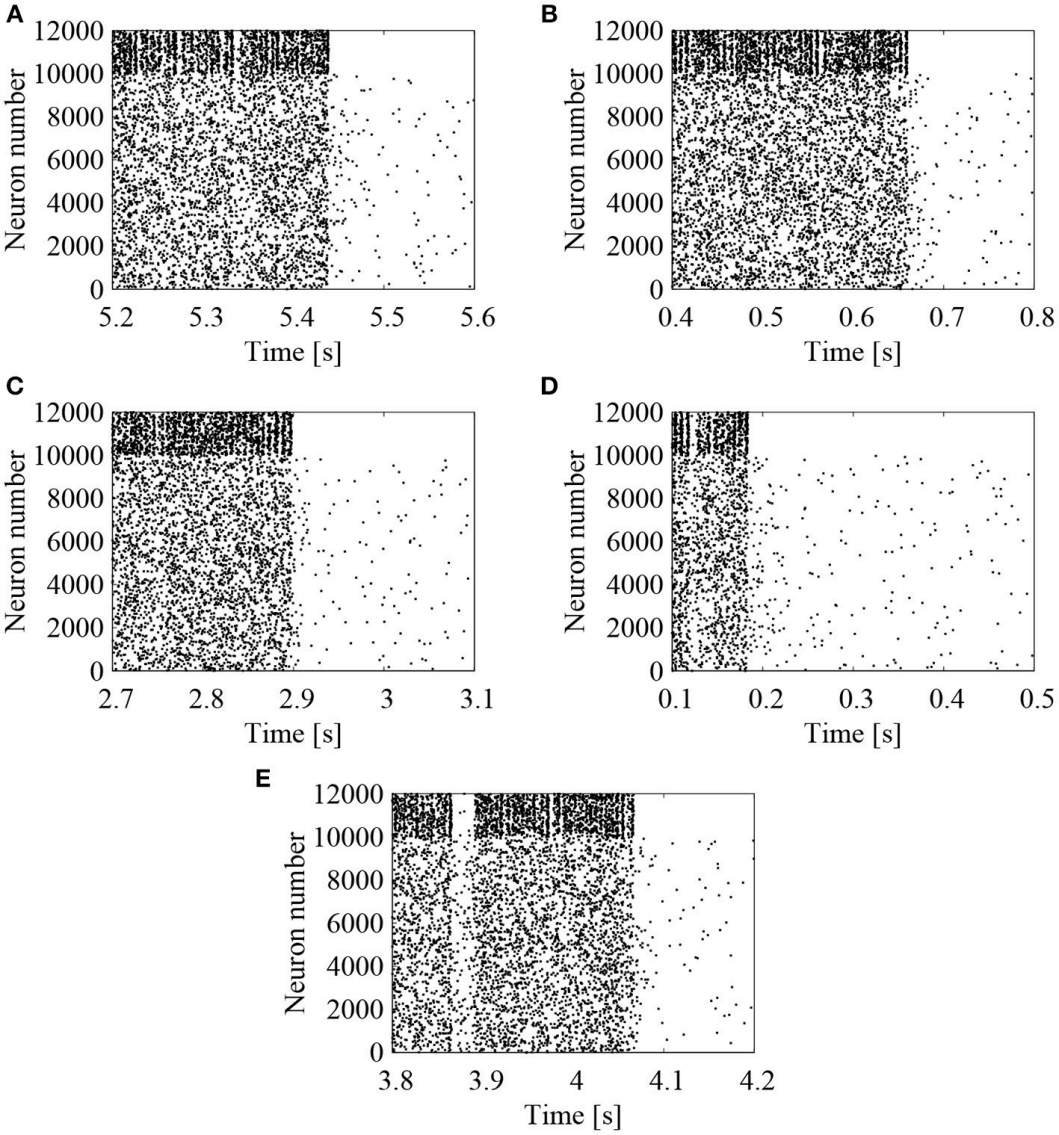
Raster plots representing the firing patterns of the cortical network model. Lognormal distribution of EPSPs was set according to Equation (3) with σ_*L*_ = 1.0. External *Poisson* spike trains were added with a frequency of 0.05 Hz. Spikes of excitatory (# 0-9999) and inhibitory (# 10000-11999) neurons are indicated by the black dots. Five realizations of stochastic simulations started from different random initial conditions are indicated in **(A)** (“1st”), **(B)** (“2nd”), **(C)** (“3rd”), **(D)** (“4th”), and **(E)** (“5th”). Note that the time intervals are different between **(A–E)**.

**Figure 3:**
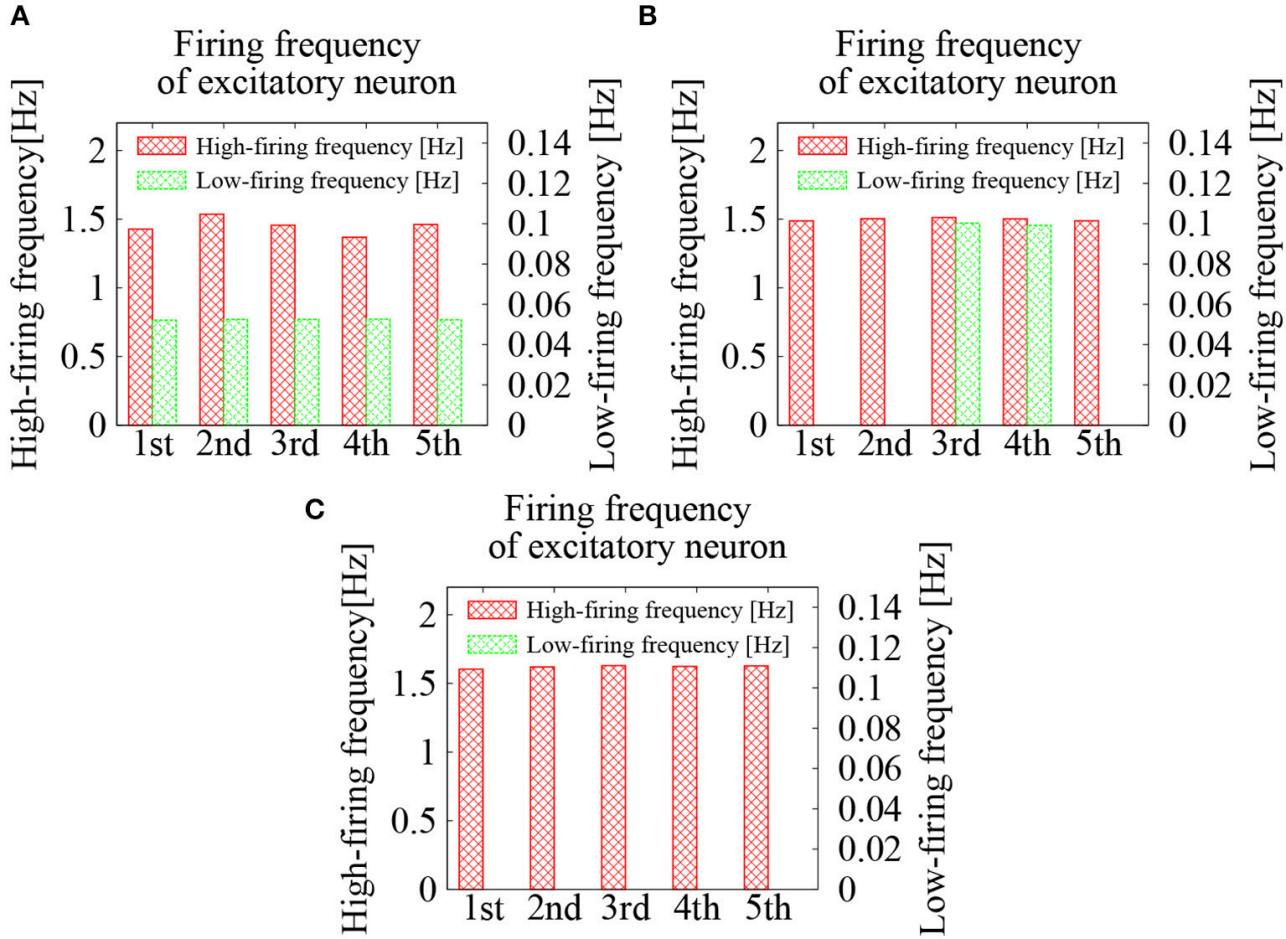
Average firing frequencies of the excitatory neurons in the cortical network model. The firing dynamics were classified into high-frequency network firing and low-frequency quiescent states, which were plotted separately. Lognormal distribution of the EPSPs was set according to Equation (3) with σ_*L*_ = 1.0. Frequencies of the external *Poisson* spike trains were 0.05 Hz in **(A)**, 0.09 Hz in **(B)**, and 0.2 Hz in **(C)**.

We first examined the effect of external *Poisson* spike trains on the cortical network model. The EPSP amplitudes between excitatory neurons were lognormally distributed with σ_*L*_ = 1.0. To activate the network firing state, relatively strong *Poisson* spikes with a frequency of 1 Hz were applied during the initial duration of 100 ms. After 100 ms, frequency of the external spike inputs was lowered to 0.05 Hz. The results are shown in the raster plots of Figures 2A–E. First, the network firings were activated by the initial application of strong external inputs. After the initial transients, the firing activities were eventually weakened and the firing level was lowered to that of the external noise. The duration, for which the high-frequency network firings last, was variable, depending upon the random initial conditions (note that the time intervals are different in Figures 2A–E). For high-frequency network firing and low-frequency quiescent states, their average firing frequencies of the excitatory neurons were comparable among different initial conditions (Figure 3A).

Next, frequency of the external spike inputs, which were given after the initial strong inputs, was increased to 0.09 Hz. The duration of the network firings was lengthened. Among 5 random initial conditions, 3 of them sustained network firing activities for more than 10 s and only 2 switched to low-frequency quiescent states before 10 s. In the case that the frequency of the external spikes was further increased to 0.2 Hz, the network firings became stable. For all 5 random initial conditions, the network firing activities lasted more than 10 s.

For the external spike inputs of 0.05, 0.09, and 0.2 Hz, average firing frequencies of the excitatory neurons during the quiescent states were slightly above those of the external inputs (see Figure 3). During the network firing activities, the average firing frequencies were around 1.5 Hz, which were independent of both initial conditions and frequency of the external inputs. The fact that these firing frequencies were much higher than those of the external inputs implies that the high-frequency activities are due to intrinsic network dynamics. The external noise merely supported the base-line level of the network activity.

### 3.2. Bistability of Low and High Frequency States

**Figure 4:**
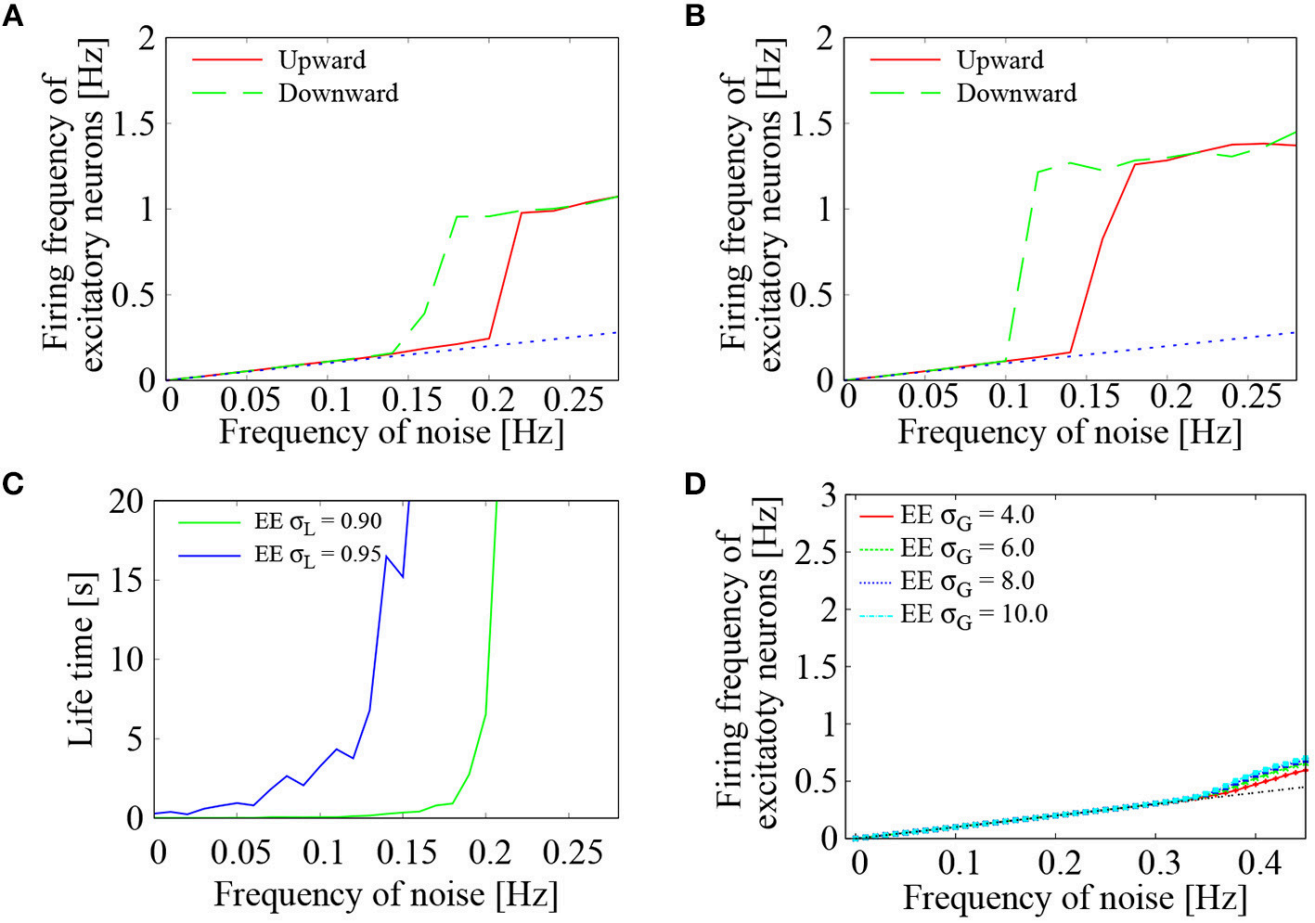
Dependence of the network firings on external noise. **(A,B)** Average firing frequency of the excitatory neurons plotted as a function of the external noise frequency. Two curves were drawn by increasing (green solid line) and decreasing (blue dotted line) the noise level. Standard deviation of the lognormally distributed EPSPs was set to σ_*L*_ = 0.90 in **(A)** and σ_*L*_ = 0.95 in **(B)**. The dashed black line corresponds to base-line frequency of the external noise. **(C)** Life-time of the network firing states was plotted as a function of the external noise frequency. Standard deviation of the lognormally distributed EPSPs was set to σ_*L*_ = 0.90 (green solid line) and σ_*L*_ = 0.95 (blue dotted line). **(D)** Case of normally distributed EPSPs. Average firing frequency of the excitatory neurons was plotted as a function of the external noise frequency. Four curves were drawn for the normal distribution with σ_*G*_ = 4.0 (red), σ_*G*_ = 6.0 (green), σ_*G*_ = 8.0 (blue), and σ_*G*_ = 10.0 (light blue). The dashed black line corresponds to base-line frequency of the external noise.

To further study the effect of external *Poisson* inputs on the network of lognormally distributed EPSPs, dependence of the firing frequency of the excitatory neurons on the frequency of noise inputs was computed in more detail. Two settings of the standard deviations, i.e., σ_*L*_ = 0.90, 0.95, were examined. In each setting, frequency of the external noise was initially set to 0 Hz. During the network simulation, the noise frequency was increased by 0.02 Hz every one second until it reached to 0.28 Hz. The noise frequency was then decreased by 0.02 Hz every one second until it reached to 0 Hz. For both increasing and decreasing frequency of the external noise, average firing frequency of the excitatory neurons was plotted in Figures 4A,B. We see a clear transition between low-frequency quiescent state and high-frequency network firing. The low-frequency states coincide with the base-line frequencies of the noise input (dashed black line), whereas the high-frequency states are clearly distinguished from them. Bistability of the two states is indicated by the hysteresis region. This is consistent with Kriener et al. (2014), who showed that saddle-node bifurcation gives rise to bistability of quiescent state and moderate firing state in a network of heavy tailed distribution of excitatory-to-excitatory synapses.

To further study the existence domain of high-frequency network firing, its life-time was computed following the procedure of Kriener et al. (2014). For each setting of the input noise frequency, strong noise with a frequency of 0.3 Hz was injected during initial 10 ms to induce high-frequency network firings. Then, the noise frequency was lowered to the setting level and the system was ran for 2 s. For different initial conditions and different realizations of the noise processes, this free-run was repeated for 30 times. From the 30 time traces, their average firing rate *r*(*t*) was obtained with a sampling time interval of 1.5 s. The averaged time trace was fitted to an exponential decay function as *r*(*t*) = *A*exp(−*t*/λ), where the estimated life-time was given by λ. As shown in Figure 4C, the life-time grew rapidly as the frequency of the external noise was increased. The critical point of the noise frequency, above which the high-frequency network firings became stable, was lowered as the standard deviation of the lognormally distributed EPSPs was increased from σ_*L*_ = 0.90 to 0.95.

To compare the lognormal EPSP distribution with *Gaussian* EPSP distribution, we simulated a network with normally distributed EPSPs $N\left(\mu_{G},\sigma_{G}^{2}\right)$. Standard deviation of the *Gaussian* distribution was varied as σ_*G*_ = 4.0, 6.0, 8.0, 10.0, while the averaged EPSP was set to be the same as that of the lognormally distributed EPSPs in the following manner. To generate realistic EPSP values, the EPSP amplitudes were restricted between 0 and 20 mV. For a given σ_*G*_, the mean parameter μ_*G*_ was determined in such a way that the probability density function *p*_*G*_(*x*) = $\exp\left[-{\left(x-\mu_{G}\right)}^{2}/2\sigma_{G}^{2}\right]$ / $\int_{0}^{20}\exp\left[-{\left(x-\mu_{G}\right)}^{2}/2\sigma_{G}^{2}\right]dx$ (0 ≤ *x* ≤ 20) yields an average EPSP equal to that of the lognormal EPSP distribution. To randomly generate EPSP values within a range between 0 and 20 mV, acceptance-rejection method (Neal, 2003) was utilized. Figure 4D shows the results. As the frequency of the noise inputs was increased, the average firing frequency of the excitatory neurons monotonously increased. In the four settings of the normally distributed EPSPs, the average firing frequency lay on the level of input noise up to 0.035 Hz. No significant frequency jump was observed. Even after the onset of network firing state, deviation of the average firing frequency from that of the input noise was rather minor. Between increasing and decreasing directions of the noise frequency, no hysteresis was observed.

### 3.3. Comparison of Onset Noise Frequency

Next, we studied onset frequency of the external noise, that is required to induce network firing state, and its dependence on the EPSP distributions. The onset point was determined as follows. First, distribution of the EPSPs and frequency of the input noise were fixed. Then, the network was simulated by supplying a strong external noise (frequency of 1 Hz) during initial duration of 100 ms. After 100 ms, frequency of the external noise was lowered to the setting level and the system was ran for 10 s. From time interval between 500 ms and 10, 100 ms, average firing frequency of the excitatory neurons was computed. This simulation was repeated for 5 different realizations of the random initial conditions and the input noise processes. Second, for each setting of the EPSP distribution, the noise frequency was increased from 0 to 0.3 Hz. The onset frequency was detected at the point, where, for all 5 realizations, the average firing frequency became larger than that of the input noise by 0.08 Hz and standard deviation of the average frequencies of the 5 realizations became less than 0.03 Hz.

**Figure 5:**
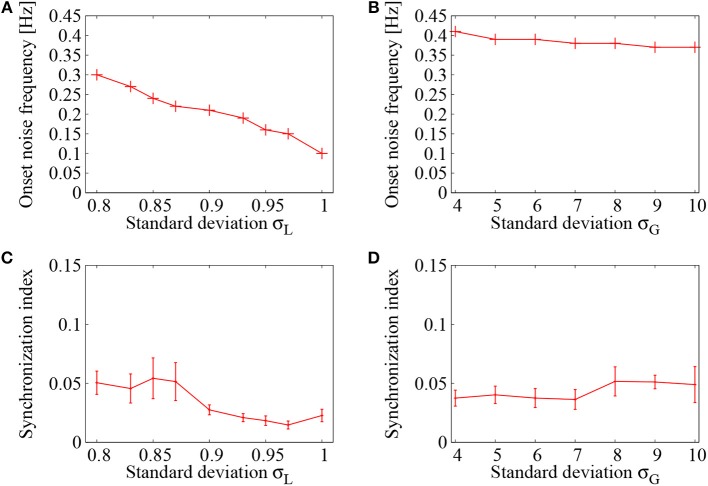
Dependence of the network firings on control parameter of the EPSP distribution. Case of lognormally distributed EPSPs **(A,C)** is compared with that of normally distributed EPSPs **(B,D)**. **(A,C)** Onset noise frequency drawn as a function of the standard deviation σ_*L*_ of lognormally distributed EPSPs in **(A)** and σ_*G*_ of normally distributed EPSPs in **(C)**. **(B,D)** Synchronization indices of the network dynamics corresponding to **(A,C)**, respectively. At each point, the error bar indicates standard deviation of 5 realizations started from different random initial conditions.

Figure 5A displays dependence of the onset noise frequency on the standard deviation σ_*L*_ of the lognormally distributed EPSPs. As the standard deviation σ_*L*_ of the lognormal distribution is increased, the onset noise frequency continues to decrease and it reaches to a value less than 0.1, that is about three times smaller than that of σ_*L*_ = 0.8. Figure 5C, on the other hand, shows dependence of the onset noise frequency on the standard deviation σ_*G*_ of the normally distributed EPSPs. Compared to the case of lognormal distribution, the onset noise frequency is much higher in the case of normal distribution. This indicates that much less amount of noise is needed to induce network firing in a network of lognormally distributed EPSPs.

As another quantity of the network dynamics, the synchronization indices corresponding to Figures 5A,B were shown in Figures 5C,D, respectively. In both cases, the synchronization index was kept within a low level (*SI* < 0.05), indicating that asynchronous firings have been realized in this region. It has been shown by Kada et al. (2016) that heterogeneity in the inhibitory-to-excitatory connections plays a key role in suppressing synchronized firing activities in a cortical network. The lognormal distribution, introduced to the inhibitory-to-excitatory connections, functioned efficiently to maintain the desynchronized firing dynamics.

### 3.4. Bernoulli Distribution

In the previous subsection, the network of lognormally distributed EPSPs has been shown to require much lower frequency of noise to induce network firing activities, compared to that of normally distributed EPSPs. This result implies that modest heterogeneity of EPSP amplitudes, i.e., the *Gaussian* distribution, is not efficient for giving rise to network firings, while the highly heterogeneous EPSP distribution, such as the lognormal distribution, works efficiently to support the firing state with less amount of noise. *Which statistical properties of the lognormal EPSP distribution are essential for maintaining the network firing activities?* One of the key feature of the lognormal distribution is that it has a long-tailed distribution with many weak and a few extremely strong synapses. As another type of such distribution, we introduce *Bernoulli* distribution of binary EPSPs. For two values of EPSP amplitudes *a*, *b* (*a*<*b*), their probabilities are defined as *P*_*a*_ = *p* and *P*_*b*_ = 1−*p*, respectively. Mean and standard deviation of the binary EPSPs are given by *m* = *ap*+*b*(1−*p*) and $\sigma_{B}=p\left(1-p\right){\left(b-a\right)}^{2}$, respectively.

**Figure 6:**
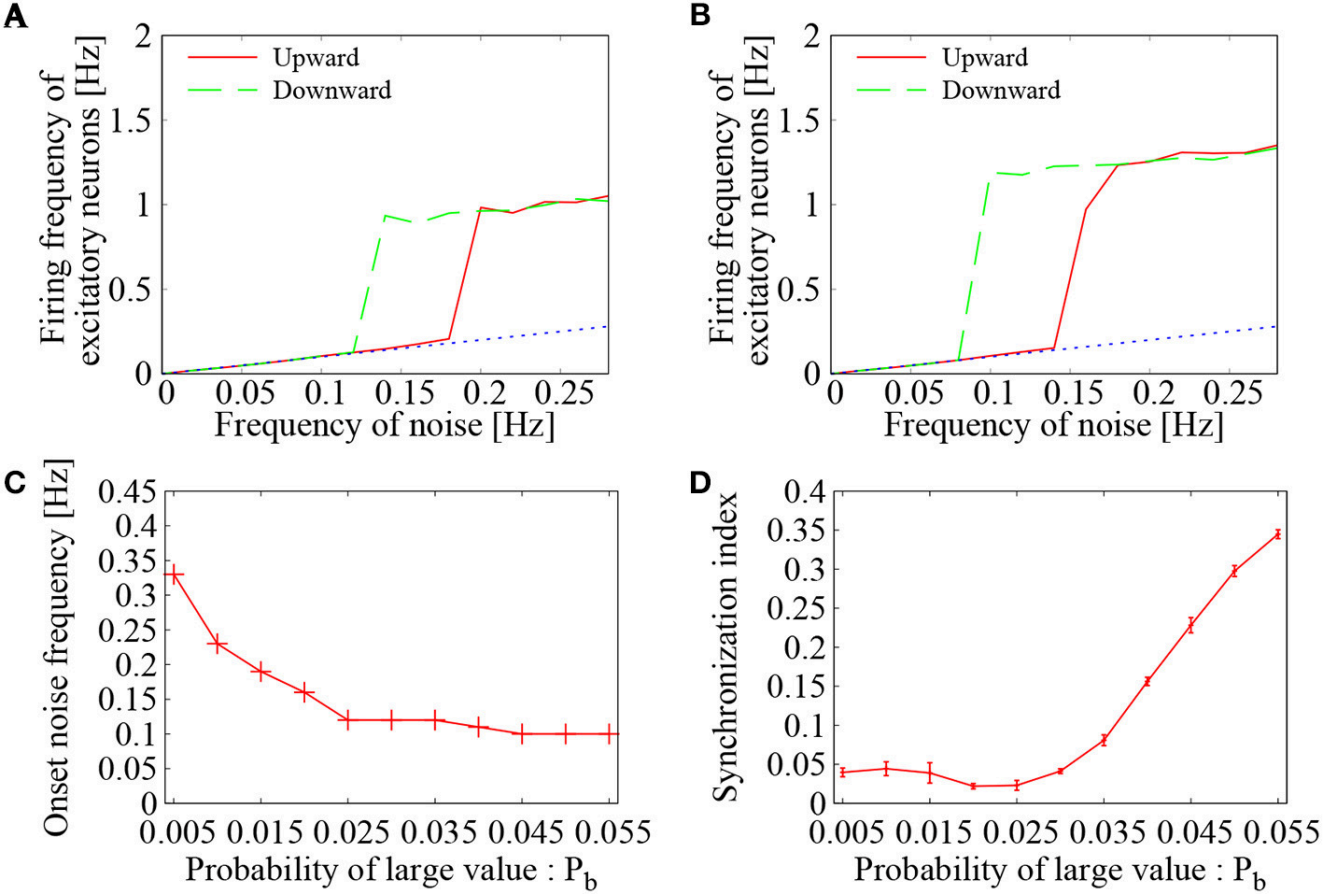
Dependence of the network firing on the probability of large value (*P*_*b*_) in *Bernoulli* distribution. **(A,B)** Mean firing frequency of the excitatory neurons as a function of the external noise frequency. Two curves were drawn by increasing (green solid line) and decreasing (blue dotted line) the noise level. In *Bernoulli* distribution of binary EPSPs, probability of large value *b* was set to *P*_*b*_ = 0.015 in **(A)** and *P*_*b*_ = 0.020 in **(B)**. The dashed black line corresponds to base-line frequency of the external noise. **(C)** Dependence of the onset noise frequency on the probability of large value (*P*_*b*_). **(D)** Synchronization index of the network dynamics corresponding to **(C)**. The error bars indicate standard deviation of 5 realizations started from different random initial conditions.

First, the effect of external *Poisson* spike trains on the network of binary EPSPs was examined. By increasing and decreasing the external noise frequency, average firing frequency of the excitatory neurons was drawn in the hysteresis plots of Figures 6A,B. The value for large EPSP amplitude was set to *b* = 9 mV, whereas its probability was set to *P*_*b*_ = 0.015 (Figure 6A) and *P*_*b*_ = 0.020 (Figure 6B). For the two settings, the mean EPSP was set to be the same as *m* = 0.9 mV. In both settings of *P*_*b*_, we see a clear transition between low-frequency quiescent state and high-frequency network firings. The low-frequency states lie on the base-line frequencies of the noise input (dashed black line), whereas the high-frequency states are distinguished from them. The hysteresis region indicates bistability of the two states. These features agree quite well with those of the network having lognormally distributed EPSPs.

Figure 6C displays dependence of the onset noise frequency on the probability *P*_*b*_ of large EPSP. As the probability *P*_*b*_ is increased, the onset noise frequency continues to decrease and reaches to a value close to 0.1 Hz. This indicates that a larger number of large EPSP amplitudes is quite helpful to give rise to network firing, when the external noise input is rather weak. On the other hand, the synchronization indices of Figure 6D show that the level of synchrony reaches to a high level (*SI*~0.35) as the probability *P*_*b*_ is increased. Too many strong synapses may create hubs in the complex network of neurons, where spike generation from such hubs leads to synchronized firings of the connected neurons. Thus, to realize asynchronous network firing, too many strong synapses may not be desired. Taking into account both the onset noise frequency and the synchronization indices, not too large, not too small, but intermediate number of large EPSPs is needed to maintain the asynchronous network firing state.

**Figure 7:**
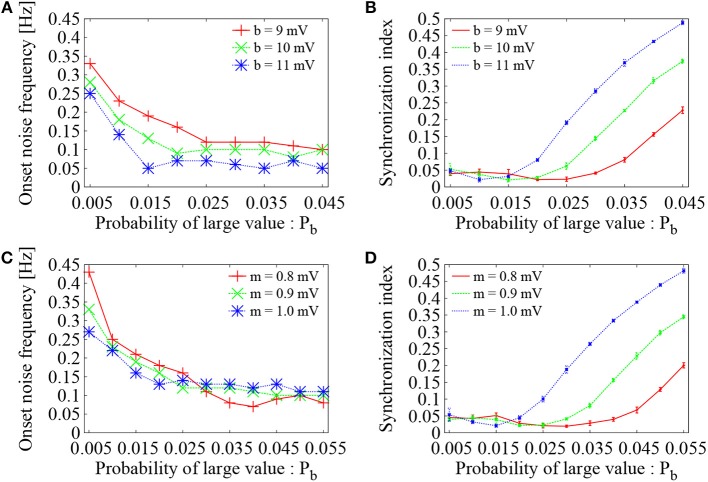
Dependence of Figure 6 on parameter setting of the *Bernoulli* distribution. **(A)** Dependence of the onset noise frequency on the probability (*P*_*b*_) of large value. The red solid line is identical to Figure 6C (*b* = 9 mV), whereas green and blue dotted lines correspond to the cases of *b* = 10 mV and *b* = 11 mV, respectively. As the probability *P*_*b*_ was varied, average of the EPSPs was set to be constant (*m* = 0.9 mV). **(B)** Synchronization index of the network dynamics corresponding to **(A)**. The error bars indicate standard deviation of 5 realizations started from different random initial conditions. **(C)** Dependence of the onset noise frequency on the probability (*P*_*b*_) of large value. Large EPSP value was set to *b* = 9 mV, whereas average of the EPSPs was kept as *m* = 0.9 mV (green dotted line), *m* = 0.8 mV (red solid line), and *m* = 1.0 mV (blue dotted line). **(D)** Synchronization index of the network dynamics corresponding to **(C)**.

To see the generality of the present analysis, dependence of the optimal configuration of the *Bernoulli* (binary) distribution on the value of large EPSP amplitude *b* was further examined. For three settings of the large EPSP amplitudes, i.e., *b* = 9 mV (red), 10 mV (green), and 11 mV (blue), the probability *P*_*b*_ was varied, while the mean EPSP was kept to be the same as *m* = 0.9 mV. As the probability *P*_*b*_ was increased, the onset noise frequency decreased and reached to a value close to 0.1 Hz or even lower (Figure 7A). The corresponding synchronization indices of Figure 7B increased to a high level as the probability *P*_*b*_ was increased. The same tendency as Figure 6 was therefore confirmed. The optimal probability of large EPSPs, at which the onset noise frequency reaches the bottom level and at the same time the synchronization index *SI* is minimized, was *P*_*b*_ = 0.025, 0.02, and 0.015 for *b* = 9, 10, and 11 mV, respectively. As the EPSP took larger values, smaller number of large EPSPs is required to maintain asynchronous firing state.

To further study the effect of the mean EPSP, three settings were considered as *m* = 0.8 mV (red), 0.9 mV (green), and 1 mV (blue). The probability *P*_*b*_ was varied, while the value of large EPSP amplitude was fixed to *b* = 9 mV. Again, as the probability *P*_*b*_ was increased, the onset noise frequency decreased (Figure 7C) and the corresponding synchronization indices increased (Figure 7D). The optimal number of large EPSPs to maintain asynchronous firing state was lowered as the mean EPSP was increased.

The present analysis based on *Bernoulli* distribution of the EPSP amplitudes is therefore general in the sense that it does not depend upon the detailed setting of the binary distribution (*b* and *m*). Neither too large nor too small numbers of large EPSPs works. There exist an optimal number of large EPSPs, which efficiently sustain the network firing activities and at the same time suppress their synchronous firings. Although there exist various types of long-tailed distributions that can represent many weak and a few extremely strong EPSPs, the *Bernoulli* model, which represents one of the simplest distribution types, suggests that the essential mechanism of sustaining the network firing activity can be elucidated by only a pair of one weak and one strong EPSP values and their balance.

## 4. Discussions

In this paper, cooperative dynamics that emerge from a network of highly heterogeneous synaptic connections and external noise inputs have been investigated. Heterogeneous distribution, typically a lognormal distribution, of excitatory-to-excitatory connection strengths have been reported experimentally (Song et al., 2005; Ikegaya et al., 2013). External noise stimuli can be thought of as representing the variability of presynaptic discharges to single cells (Destexhe and Contreras, 2006) or as endogenously active cells that stimulate the neighboring quiescent cells (Latham et al., 2000a,b). As the combined effect, the noise works efficiently to activate intrinsic network dynamics to give rise to asynchronous firings. Comparative study with *Gaussian* distribution as well as *Bernoulli* distribution revealed that weak-dense and sparse-strong connections are the key feature of the synapses that support the stable firing activity. Our finding is consistent with the theoretical study of the autonomous network of lognormal EPSP distribution, which was shown to be capable of sustaining spontaneous firings without external stimulus (Teramae et al., 2012; Kriener et al., 2014). The present study extends such idea to the case of non-autonomous network having external stimuli, where the long-tailed EPSP distribution reduces the noise level needed for the onset of network firings. In the sense that stochastic noise is ubiquitous in neural systems, this extension is of essential importance to incorporate the lognormal network to more realistic neurophysiological situation. Since synaptic noise requires an energy resource (Laughlin et al., 1998; Attwell and Laughlin, 2001; Lennie, 2003), it is biologically more efficient to maintain the firing activities with a few support from such expensive resource. The fact that the network of lognormally distributed EPSPs effectively reduced the noise level compared to that of normally distributed EPSPs indicates that highly heterogeneous distribution of excitatory-to-excitatory connection strengths can be advantageous for maintaining network firing activity in an efficient and robust fashion.

It should be noted that energy expenditure in the cortical system provides an intriguing issue. Not only the energy spent for spike generations (Laughlin et al., 1998; Attwell and Laughlin, 2001; Lennie, 2003; Harris et al., 2012), resting potentials (Harris and Attwell, 2012), and other electrical activities, but also metabolic energy spent for development of synapses (Karbowski, 2012) and their maintenance (Bezprozvanny and Hiesinger, 2013; Tononi and Cirelli, 2014) have been recently discussed. The advantage of having few but very strong synapses should be considered carefully by taking into account the maintenance cost of such strong synapses.

In the present study, the external noise inputs to individual neurons have been assumed to be independent from each other. Considering the real neurophysiology, where synaptic projections are overlapped among neurons and the neurons may receive common stimuli from the same external sources, it would be more realistic to assume a certain level of correlation in the noise inputs. It is therefore an important future study to deal with the case that the external noise is spatially correlated. How to separate synaptic noise from irregular firings that originate from intrinsic network dynamics provides also an important open problem to quantitatively examine our modeling study in experimental data.

Interesting application of the present study is to model the transition of cortical neurons between Up (depolarized) and Down (hyperpolarized) states. Stimulus-dependent transitions between the Up and Down states have been reported in cat visual cortex (Anderson et al., 2000). Single neuron membrane potentials in slice preparations also show spontaneous transition between them (Cossart et al., 2003; Shu et al., 2003b), where intrinsic network dynamics is considered to play an important role (Cossart et al., 2003). Such transition has been modeled as bistable Up and Down states, embedded in a network of excitatory and inhibitory neurons (Wilson and Cowan, 1972), where the switching can be triggered, e.g., by synaptic depression (Bazhenov et al., 2002). Synaptic noise can lead to random transitions between the basins of Up–Down states (Holcman and Tsodyks, 2006). It should be of great interest to examine how the lognormal network combined with the noise stimuli may model such Up–Down transitions.

## Author Contributions

IT and JT designed the study. JT, HK, and IT discussed the results. HK performed the model simulations. HK and IT analyzed the data. IT and JT wrote the text.

### Conflict of Interest Statement

The authors declare that the research was conducted in the absence of any commercial or financial relationships that could be construed as a potential conflict of interest.

## References

[B1] Anderson J. Lampl I. Reichova I. Carandini M. Ferster D. (2000). Stimulus dependence of two-state fluctuations of membrane potential in cat visual cortex. Nat. Neurosci. 3, 617. 10.1038/75797 10816319

[B2] Arieli A. Sterkin A. Grinvald A. Aertsen A. (1996). Dynamics of ongoing activity: explanation of the large variability in evoked cortical responses. Science 273, 1868–1871. 10.1126/science.273.5283.1868 8791593

[B3] Aronov D. (2003). Fast algorithm for the metric-space analysis of simultaneous responses of multiple single neurons. J. Neurosci. Methods 124, 175–179. 10.1016/S0165-0270(03)00006-2 12706847

[B4] Attwell D. Laughlin S. B. (2001). An energy budget for signaling in the grey matter of the brain. J. Cereb. Blood Flow Metab. 21, 1133–1145. 10.1097/00004647-200110000-00001 11598490

[B5] Avermann M. Tomm C. Mateo C. Gerstner W. Petersen C. C. (2012). Microcircuits of excitatory and inhibitory neurons in layer 2/3 of mouse barrel cortex. J. Neurophysiol. 107, 3116–3134. 10.1152/jn.00917.2011 22402650

[B6] Bazhenov M. Timofeev I. Steriade M. Sejnowski T. J. (2002). Model of thalamocortical slow-wave sleep oscillations and transitions to activated states. J. Neurosci. 22, 8691–8704. 10.1523/JNEUROSCI.22-19-08691.2002 12351744 PMC6757797

[B7] Bezprozvanny I. Hiesinger P. R. (2013). The synaptic maintenance problem: membrane recycling, ca 2+ homeostasis and late onset degeneration. Mol. Neurodegener. 8:23 10.1186/1750-1326-8-23 23829673 PMC3708831

[B8] Bremner F. Baker J. Stephens J. (1991). Variation in the degree of synchronization exhibited by motor units lying in different finger muscles in man. J. Physiol. 432, 381–399. 10.1113/jphysiol.1991.sp018390 1886060 PMC1181331

[B9] Brunel N. (2000). Dynamics of sparsely connected networks of excitatory and inhibitory spiking neurons. J. Comput. Neurosci. 8, 183–208. 10.1023/A:1008925309027 10809012

[B10] Buzsáki G. Mizuseki K. (2014). The log-dynamic brain: how skewed distributions affect network operations. Nat. Rev. Neurosci. 15, 264–278. 10.1038/nrn3687 24569488 PMC4051294

[B11] Chapeton J. Fares T. LaSota D. Stepanyants A. (2012). Efficient associative memory storage in cortical circuits of inhibitory and excitatory neurons. Proc. Natl. Acad. Sci. 109, E3614–E3622. 10.1073/pnas.1211467109 23213221 PMC3529061

[B12] Compte A. (2006). Computational and *in vitro* studies of persistent activity: edging towards cellular and synaptic mechanisms of working memory. Neuroscience 139, 135–151. 10.1016/j.neuroscience.2005.06.011 16337341

[B13] Cossart R. Aronov D. Yuste R. (2003). Attractor dynamics of network up states in the neocortex. Nature 423, 283. 10.1038/nature01614 12748641

[B14] Datta A. Stephens J. (1990). Synchronization of motor unit activity during voluntary contraction in man. J. Physiol. 422, 397–419. 10.1113/jphysiol.1990.sp017991 2352185 PMC1190139

[B15] Destexhe A. Contreras D. (2006). Neuronal computations with stochastic network states. Science 314, 85–90. 10.1126/science.1127241 17023650

[B16] Destexhe A. Rudolph M. Fellous J.-M. Sejnowski T. J. (2001). Fluctuating synaptic conductances recreate *in vivo*-like activity in neocortical neurons. Neuroscience 107, 13–24. 10.1016/S0306-4522(01)00344-X 11744242 PMC3320220

[B17] Destexhe A. Rudolph M. Paré D. (2003). The high-conductance state of neocortical neurons *in vivo*. Nat. Rev. Neurosci. 4, 739–751. 10.1038/nrn1198 12951566

[B18] Engel A. K. König P. Kreiter A. K. Singer W. (1991). Interhemispheric synchronization of oscillatory neuronal responses in cat visual cortex. Science 1177–1179. 10.1126/science.252.5009.1177 2031188

[B19] Faisal A. A. Selen L. P. Wolpert D. M. (2008). Noise in the nervous system. Nat. Rev. Neurosci. 9, 292–303. 10.1038/nrn2258 18319728 PMC2631351

[B20] Fuster J. M. (1995). Memory in the Cerebral Cortex. Cambridge, MA: The MIT Press.

[B21] Gray C. M. König P. Engel A. K. Singer W. (1989). Oscillatory responses in cat visual cortex exhibit inter-columnar synchronization which reflects global stimulus properties. Nature 338, 334. 10.1038/338334a0 2922061

[B22] Gross G. W. Williams A. N. Lucas J. H. (1982). Recording of spontaneous activity with photoetched microelectrode surfaces from mouse spinal neurons in culture. J. Neurosci. Methods 5, 13–22. 10.1016/0165-0270(82)90046-2 7057675

[B23] Harris J. J. Attwell D. (2012). The energetics of cns white matter. J. Neurosci. 32, 356–371. 10.1523/JNEUROSCI.3430-11.2012 22219296 PMC3272449

[B24] Harris J. J. Jolivet R. Attwell D. (2012). Synaptic energy use and supply. Neuron 75, 762–777. 10.1016/j.neuron.2012.08.019 22958818

[B25] Holcman D. Tsodyks M. (2006). The emergence of up and down states in cortical networks. PLoS Comput. Biol. 2:e23. 10.1371/journal.pcbi.0020023 16557293 PMC1409813

[B26] Holmgren C. Harkany T. Svennenfors B. Zilberter Y. (2003). Pyramidal cell communication within local networks in layer 2/3 of rat neocortex. J. Physiol. 551, 139–153. 10.1113/jphysiol.2003.044784 12813147 PMC2343144

[B27] Hromádka T. DeWeese M. R. Zador A. M. (2008). Sparse representation of sounds in the unanesthetized auditory cortex. PLoS Biol. 6:e16. 10.1371/journal.pbio.0060016 18232737 PMC2214813

[B28] Ikegaya Y. Sasaki T. Ishikawa D. Honma N. Tao K. Takahashi N.. (2013). Interpyramid spike transmission stabilizes the sparseness of recurrent network activity. Cereb. Cortex 23, 293–304. 10.1093/cercor/bhs006 22314044

[B29] Jiang X. Shen S. Cadwell C. R. Berens P. Sinz F. Ecker A. S.. (2015). Principles of connectivity among morphologically defined cell types in adult neocortex. Science 350:aac9462. 10.1126/science.aac9462 26612957 PMC4809866

[B30] Kada H. Teramae J.-N. Tokuda I. T. (2016). Effective suppression of pathological synchronization in cortical networks by highly heterogeneous distribution of inhibitory connections. Front. Comput. Neurosci. 10:109. 10.3389/fncom.2016.00109 27803659 PMC5067923

[B31] Karbowski J. (2012). Approximate invariance of metabolic energy per synapse during development in mammalian brains. PLoS ONE 7:e33425. 10.1371/journal.pone.0033425 22479396 PMC3314021

[B32] Kenet T. Bibitchkov D. Tsodyks M. Grinvald A. Arieli A. (2003). Spontaneously emerging cortical representations of visual attributes. Nature 425, 954–956. 10.1038/nature02078 14586468

[B33] Kreuz T. Chicharro D. Andrzejak R. G. Haas J. S. Abarbanel H. D. (2009). Measuring multiple spike train synchrony. J. Neurosci. Methods 183, 287–299. 10.1016/j.jneumeth.2009.06.039 19591867

[B34] Kreuz T. Haas J. S. Morelli A. Abarbanel H. D. Politi A. (2007). Measuring spike train synchrony. J. Neurosci. Methods 165, 151–161. 10.1016/j.jneumeth.2007.05.031 17628690

[B35] Kriener B. Enger H. Tetzlaff T. Plesser H. E. Gewaltig M.-O. Einevoll G. T. (2014). Dynamics of self-sustained asynchronous-irregular activity in random networks of spiking neurons with strong synapses. Front. Comput. Neurosci. 8:136. 10.3389/fncom.2014.00136 25400575 PMC4214205

[B36] Kumar A. Rotter S. Aertsen A. (2010). Spiking activity propagation in neuronal networks: reconciling different perspectives on neural coding. Nat. Rev. Neurosci. 11, 615–627. 10.1038/nrn2886 20725095

[B37] Kumar A. Schrader S. Aertsen A. Rotter S. (2008). The high-conductance state of cortical networks. Neural Comput. 20, 1–43. 10.1162/neco.2008.20.1.1 18044999

[B38] Latham P. Richmond B. Nelson P. Nirenberg S. (2000a). Intrinsic dynamics in neuronal networks. i. theory. J. Neurophysiol. 83, 808–827. 10.1152/jn.2000.83.2.808 10669496

[B39] Latham P. Richmond B. Nirenberg S. Nelson P. (2000b). Intrinsic dynamics in neuronal networks. ii. experiment. J. Neurophysiol. 83, 828–835. 10.1152/jn.2000.83.2.828 10669497

[B40] Laughlin S. B. de Ruyter van Steveninck R. R. Anderson J. C. (1998). The metabolic cost of neural information. Nat. Neurosci. 1, 36. 10.1038/236 10195106

[B41] Lefort S. Tomm C. Sarria J.-C. F. Petersen C. C. (2009). The excitatory neuronal network of the c2 barrel column in mouse primary somatosensory cortex. Neuron 61, 301–316. 10.1016/j.neuron.2008.12.020 19186171

[B42] Lennie P. (2003). The cost of cortical computation. Curr. Biol. 13, 493–497. 10.1016/S0960-9822(03)00135-0 12646132

[B43] Logigian E. L. Wierzbicka M. M. Bruyninckx F. Wiegner A. W. Shahahi B. T. Young R. R. (1988). Motor unit synchronization in physiologic, enhanced physiologic, and voluntary tremor in man. Ann. Neurol. 23, 242–250. 10.1002/ana.410230306 2967666

[B44] London M. Roth A. Beeren L. Häusser M. Latham P. E. (2010). Sensitivity to perturbations *in vivo* implies high noise and suggests rate coding in cortex. Nature 466, 123–127. 10.1038/nature09086 20596024 PMC2898896

[B45] Mao B.-Q. Hamzei-Sichani F. Aronov D. Froemke R. C. Yuste R. (2001). Dynamics of spontaneous activity in neocortical slices. Neuron 32, 883–898. 10.1016/S0896-6273(01)00518-9 11738033

[B46] Marom S. Shahaf G. (2002). Development, learning and memory in large random networks of cortical neurons: lessons beyond anatomy. Q. Rev. Biophys. 35, 63–87. 10.1017/S0033583501003742 11997981

[B47] Miles R. Wong R. (1984). Unitary inhibitory synaptic potentials in the guinea-pig hippocampus *in vitro*. J. Physiol. 356, 97–113. 10.1113/jphysiol.1984.sp015455 6097680 PMC1193154

[B48] Mizuseki K. Buzsáki G. (2013). Preconfigured, skewed distribution of firing rates in the hippocampus and entorhinal cortex. Cell Rep. 4, 1010–1021. 10.1016/j.celrep.2013.07.039 23994479 PMC3804159

[B49] Neal R. M. (2003). Slice sampling. Ann. Stat. 31, 705–741. 10.1214/aos/1056562461

[B50] Nordstrom M. A. Fuglevand A. Enoka R. (1992). Estimating the strength of common input to human motoneurons from the cross-correlogram. J. Physiol. 453, 547–574. 10.1113/jphysiol.1992.sp019244 1464844 PMC1175573

[B51] Plenz D. Aertsen A. (1996). Neural dynamics in cortex-striatum co-cultures spatiotemporal characteristics of neuronal activity. Neuroscience 70, 893–924. 10.1016/0306-4522(95)00405-X 8848173

[B52] Quian Quiroga R. Q. Kreuz T. Grassberger P. (2002). Event synchronization: a simple and fast method to measure synchronicity and time delay patterns. Phys. Rev. E 66:041904. 10.1103/PhysRevE.66.041904 12443232

[B53] van Rossum M. (2001). A novel spike distance. Neural Comput. 13, 751–763. 10.1162/089976601300014321 11255567

[B54] Shadlen M. N. Newsome W. T. (1994). Noise, neural codes and cortical organization. Curr. Opin. Neurobiol. 4, 569–579. 10.1016/0959-4388(94)90059-0 7812147

[B55] Shu Y. Hasenstaub A. Badoual M. Bal T. McCormick D. A. (2003a). Barrages of synaptic activity control the gain and sensitivity of cortical neurons. J. Neurosci. 23, 10388–10401. 10.1523/JNEUROSCI.23-32-10388.2003 14614098 PMC6741011

[B56] Shu Y. Hasenstaub A. McCormick D. A. (2003b). Turning on and off recurrent balanced cortical activity. Nature 423, 288. 10.1038/nature01616 12748642

[B57] Softky W. R. Koch C. (1993). The highly irregular firing of cortical cells is inconsistent with temporal integration of random epsps. J. Neurosci. 13, 334–350. 10.1523/JNEUROSCI.13-01-00334.1993 8423479 PMC6576320

[B58] Song S. Sjöström P. J. Reigl M. Nelson S. Chklovskii D. B. (2005). Highly nonrandom features of synaptic connectivity in local cortical circuits. PLoS Biol. 3:e68. 10.1371/journal.pbio.0030068 15737062 PMC1054880

[B59] Steriade M. Timofeev I. Grenier F. (2001). Natural waking and sleep states: a view from inside neocortical neurons. J. Neurophysiol. 85, 1969–1985. 10.1152/jn.2001.85.5.1969 11353014

[B60] Stiefel K. M. Englitz B. Sejnowski T. J. (2013). Origin of intrinsic irregular firing in cortical interneurons. Proc. Natl. Acad. Sci. U.S.A. 110, 7886–7891. 10.1073/pnas.1305219110 23610409 PMC3651468

[B61] Tamás G. Somogyi P. Buhl E. H. (1998). Differentially interconnected networks of gabaergic interneurons in the visual cortex of the cat. J. Neurosci. 18, 4255–4270. 10.1523/JNEUROSCI.18-11-04255.1998 9592103 PMC6792813

[B62] Teramae J.-N. Tsubo Y. Fukai T. (2012). Optimal spike-based communication in excitable networks with strong-sparse and weak-dense links. Sci. Rep. 2:485. 10.1038/srep00485 22761993 PMC3387577

[B63] Timofeev I. Grenier F. Bazhenov M. Sejnowski T. Steriade M. (2000). Origin of slow cortical oscillations in deafferented cortical slabs. Cereb. Cortex 10, 1185–1199. 10.1093/cercor/10.12.1185 11073868

[B64] Tomko G. J. Crapper D. R. (1974). Neuronal variability: non-stationary responses to identical visual stimuli. Brain Res. 79, 405–418. 10.1016/0006-8993(74)90438-7 4422918

[B65] Tononi G. Cirelli C. (2014). Sleep and the price of plasticity: from synaptic and cellular homeostasis to memory consolidation and integration. Neuron 81, 12–34. 10.1016/j.neuron.2013.12.025 24411729 PMC3921176

[B66] Tsodyks M. Kenet T. Grinvald A. Arieli A. (1999). Linking spontaneous activity of single cortical neurons and the underlying functional architecture. Science 286, 1943–1946. 10.1126/science.286.5446.1943 10583955

[B67] van Vreeswijk C. Sompolinsky H. (1996). Chaos in neuronal networks with balanced excitatory and inhibitory activity. Science 274, 1724–1726. 10.1126/science.274.5293.1724 8939866

[B68] Victor J. D. Purpura K. P. (1996). Nature and precision of temporal coding in visual cortex: a metric-space analysis. J. Neurophysiol. 76, 1310–1326. 10.1152/jn.1996.76.2.1310 8871238

[B69] Vogels T. P. Abbott L. F. (2005). Signal propagation and logic gating in networks of integrate-and-fire neurons. J. Neurosci. 25, 10786–10795. 10.1523/JNEUROSCI.3508-05.2005 16291952 PMC6725859

[B70] Wang X. J. (2002). Probabilistic decision making by slow reverberation in cortical circuits. Neuron 36, 955–968 10.1016/S0896-6273(02)01092-9 12467598

[B71] Wilson C. J. Kawaguchi Y. (1996). The origins of two-state spontaneous membrane potential fluctuations of neostriatal spiny neurons. J. Neurosci. 16, 2397–2410. 10.1523/JNEUROSCI.16-07-02397.1996 8601819 PMC6578540

[B72] Wilson H. R. Cowan J. D. (1972). Excitatory and inhibitory interactions in localized populations of model neurons. Biophys. J. 12, 1–24. 10.1016/S0006-3495(72)86068-5 4332108 PMC1484078

